# Supplementary material for: Eternal non-Markovianity: from random unitary to Markov chain realisations
Source: Sci Rep. 2017 Jul 25;7:6379. doi: 10.1038/s41598-017-06059-5 (PMC5527059; doi:10.1038/s41598-017-06059-5)
Supplement: Supplementary file 1 — Supplementary Information [file 41598_2017_6059_MOESM1_ESM.pdf]

# SUPPLEMENTARY INFORMATION:
# Eternal non-Markovianity: from random unitary to Markov chain realisations

Nina Megier,[1,*] Dariusz Chruściński,[2] Jyrki Piilo,[3] and Walter T. Strunz[1]

[1] *Institut für Theoretische Physik, Technische Universität Dresden, D-01062 Dresden, Germany*
[2] *Institute of Physics, Faculty of Physics, Astronomy and Informatics, Nicolaus Copernicus University, Grudziącka 5/7, 87-100 Toruń, Poland*
[3] *Turku Centre of Quantum Physics, Department of Physics and Astronomy, University of Turku, FI-20014 Turun yliopisto, Finland*

[*] nina.megier@tu-dresden.de

## I. RANDOM UNITARY DYNAMICS

The propagator of the Schrödinger equation (10) of the main text, defined through $\psi(t) = U_\xi(t;0)\psi(0)$ is

$$U_\xi(t;0) = \exp\left(-i\int_0^t \xi(s)ds\sigma_\alpha\right) = \cos(\int_0^t \xi(s)ds)\mathbb{1} - i\sin(\int_0^t \xi(s)ds)(\vec{n}\vec{\sigma}_\alpha). \tag{S.1}$$

We have $\langle\!\langle e^{2i\int_0^t \xi(s)ds}\rangle\!\rangle_\xi = e^{-2\langle\!\langle(\int_0^t \xi(s)ds)^2\rangle\!\rangle_\xi} = e^{-2t}$, such that

$$\langle\!\langle \cos(2\int_0^t \xi(s)ds)\rangle\!\rangle_\xi = e^{-2t}, \tag{S.2}$$

$$\langle\!\langle \sin(2\int_0^t \xi(s)ds)\rangle\!\rangle_\xi = 0.$$

We obtain the density operator from an average over the noise $\xi(t)$. To obtain Eq. (12) of the main text we use $\cos^2(\Phi/2) = \frac{1}{2}(1+\cos\Phi)$, $\sin^2(\Phi/2) = \frac{1}{2}(1-\cos\Phi)$, $\sin(\Phi/2)\cos(\Phi/2) = \sin(\Phi)/2$ and the ensemble averages (S.2).

## II. CALCULATION OF DECOHERENCE RATES

We write our density operator with a Bloch vector:

$$\rho(t) = \frac{1}{2}(\mathbb{1} + \vec{r}(t)\cdot\vec{\sigma}) = \frac{1}{2}(\mathbb{1} + \sum_{k=1}^{3} r_k(t)\sigma_k). \tag{S.3}$$

Plugging in this representation in Eq. (5) of the main text and comparing with the derivation of Eq. (15) we get:

$$r_1(t)(\gamma_2(t) + \gamma_3(t)) = 2\mathrm{e}^{-2t}(x_2 + x_3)r_1(0) \tag{S.4}$$

Using a relation between $r_1(t)$ and $r_1(0)$ following from Eq. (15):

$$r_1(t) = r_1(0)(x_1 + \mathrm{e}^{-2t}(x_2 + x_3)), \tag{S.5}$$

one obtains from (S.4) and analogue calculations for $r_2(t), r_3(t)$ the time dependent decoherence rates (19) of the main text.

## III. FORM OF THE ASYMPTOTIC AREA

We can parametrize $x_1, x_2, x_3$ in a following way:

$$\vec{r}(x,y) = \begin{pmatrix} x_1(x,y) \\ x_2(x,y) \\ x_3(x) \end{pmatrix} = \begin{pmatrix} \frac{1}{2}(1+\sqrt{1-4y})(1-x) \\ \frac{1}{2}(1-\sqrt{1-4y})(1-x) \\ x \end{pmatrix}, \tag{S.6}$$

with $0 \le x \le 1$, $-\frac{2x}{(1-x)^2} \le y \le \frac{1}{4}$. The vector $\vec{r}(x,y)$ is pointing from the origin of the coordinate system toward a point on the curve separating the asymptotic parameter area (asymptotic curve) - see Figure S.1.
We are looking for an asymptotic curve with $\gamma_3(t) = 0$, for which the equation

$$0 = (x_2 + x_3)x_2x_3 + (x_3 + x_1)x_1x_3 - (x_1 + x_2)x_1x_2$$

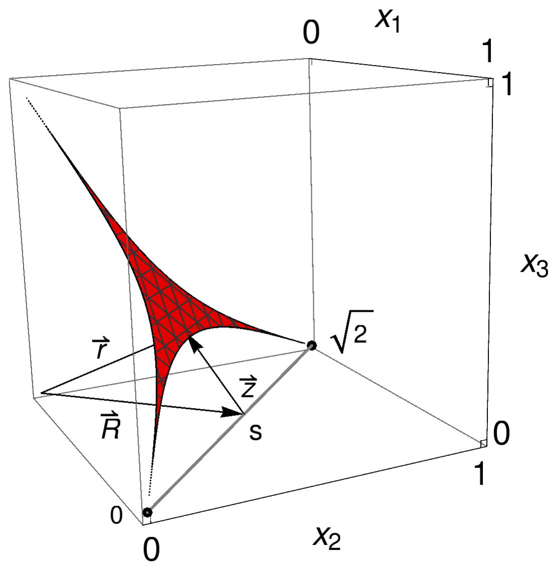


FIG. S.1. Calculating the area of the asymptotic area with a help of new vectors $\vec{R}(s)$, $\vec{z}(z)$.

is satisfied.
In terms of the new variables it is equivalent to an equation:

$$x^2y + x - y = 0,$$

which describes some Newton's cubic curve.
The solution is:

$$y(x) = \frac{x}{1-x^2},$$

with $0 \leq x \leq -2+\sqrt{5}$, $0 \leq y \leq \frac{1}{4}$.
To calculate the area of the asymptotic area we change to other variables $s$, $z$. We introduce vectors: $\vec{R}(s)$ being parallel to the $x_1 - x_2$ surface and $\vec{z}(z)$ parallel to the defining triangle, with $\vec{r}(s,z) = \vec{R}(s) + \vec{z}(z)$ - see Fig. S.1. The variable $s$ measures the distance between the point $(x_1, x_2, x_3) = (1, 0, 0)$ and the final point of the vector $\vec{R}(s)$, when the initial point is the origin of the coordinate system. The variable $z$ is the length of vector $\vec{z}$. Writing the vector $\vec{r}(s,z)$ as the sum of the $\vec{R}(s)$ and $\vec{z}(z)$:

$$\vec{r}(s,z) = \vec{R}(s) + \vec{z}(z) = \begin{pmatrix} 1 - \frac{s}{\sqrt{2}} \\ \frac{s}{\sqrt{2}} \\ 0 \end{pmatrix} + \frac{z}{\sqrt{6}} \begin{pmatrix} -1 \\ -1 \\ 2 \end{pmatrix}, \tag{S.7}$$

we get from (S.6) and (S.7):

$$s(x) = \frac{1}{\sqrt{2}} \left( 1 - (1-x)\sqrt{1 - \frac{4x}{1-x^2}} \right).$$

The asymptotic area equals:

$$A_{\text{CP-div}} = A_{\text{tot}} - 6 \int_0^{\frac{\sqrt{2}}{2}} ds z(s) = A_{\text{tot}} - 6 \int_0^{\sqrt{5}-2} dx \frac{ds(x)}{dx} z(x)$$

$$= A_{\text{tot}} - 6 \frac{\sqrt{6}}{2\sqrt{2}} \int_0^{\sqrt{5}-2} dx \frac{(3-3x-3x^2-x^3)x}{\sqrt{1-\frac{4x}{1-x^2}}(1-x^2)(1+x)}$$

and consequently one gets

$$\frac{A_{\text{CP-div}}}{A_{\text{tot}}} \approx 0.130594.$$

## IV. PAULI MASTER EQUATION

The dynamics by Eq. (5) of the main text can be represented by the following rate equation:

$$\dot{p}_0(t) = p_1(t) - x_1 p_0(t) + p_2(t) - x_2 p_0(t) + p_3(t) - x_3 p_0(t) = -p_0(t) + p_1(t) + p_2(t) + p_3(t), \tag{S.8}$$

$$\dot{p}_1(t) = x_1 p_0(t) - p_1(t), \tag{S.9}$$

$$\dot{p}_2(t) = x_2 p_0(t) - p_2(t), \tag{S.10}$$

$$\dot{p}_3(t) = x_3 p_0(t) - p_3(t). \tag{S.11}$$

From (S.9),(S.10),(S.11) we get:

$$p_1^h(t) = p_2^h(t) = p_3^h(t) = \mathrm{e}^{-t}.$$

With an ansatz: $p_k(t) = C_k(t) p_k^h(t)$ one obtains (l=1,2,3):

$$p_k(t) = \mathrm{e}^{-t}\left(x_k \int_0^t p_0(s)\mathrm{e}^s ds + C_k\right) = \frac{x_k}{x_l} p_l(t) + \mathrm{e}^{-t}\left(C_k - \frac{x_k}{x_l} C_l\right).$$

Fixing: $C_1 = C_2 = C_3 = 0$ (which corresponds to $p_1(0) = p_2(0) = p_3(0) = 0$) we obtain:

$$p_k(t) = \frac{x_k}{x_l} p_l(t).$$

Adding (S.8),(S.9),(S.10),(S.11) we get:

$$\dot{p}_0(t) = -\dot{p}_1(t) - \dot{p}_2(t) - \dot{p}_3(t) = -\dot{p}_1(t)\left(1 + \frac{x_2}{x_1} + \frac{x_3}{x_1}\right) = -\frac{1}{x_1}\dot{p}_1(t).$$

With initial condition $p_0(0) = 1$ we find:

$$p_0(t) = -\frac{1}{x_1} p_1(t) + 1,$$

from which together with (S.9) we conclude:

$$\dot{p}_1(t) = -p_1(t) + x_1 - p_1 \qquad \Rightarrow \qquad p_1(t) = \frac{x_1}{2}\left(1 - \mathrm{e}^{-2t}\right),$$

etc. and consequently Eq. (27) of the main text follows.

## V. "NON-MARKOVIAN" FORMULATION OF DYNAMICS

It is not clear that Eq. (31) of the main text defines a legitimate master equation (we only know that one particular trajectory passing through $(1,0,0,0)^T$ corresponds to the jump process of Fig. 2 of the main text). Let us observe that

$$\begin{aligned} A(t) &:= \frac{1}{2}\begin{pmatrix} -\gamma_0(t) & \gamma_1(t) & \gamma_2(t) & \gamma_3(t) \\ \gamma_1(t) & -\gamma_0(t) & \gamma_3(t) & \gamma_2(t) \\ \gamma_2(t) & \gamma_3(t) & -\gamma_0(t) & \gamma_1(t) \\ \gamma_3(t) & \gamma_2(t) & \gamma_1(t) & -\gamma_0(t) \end{pmatrix} \\ &= \gamma_1(t)a_1 + \gamma_2(t)a_2 + \gamma_3(t)a_3. \end{aligned}$$

One checks

$$[a_k, a_l] = 0,$$

and accordingly $A(t)$ provides a commutative family of generators. The corresponding solution for the map $T(t)$ satisfying

$$\frac{d}{dt}T(t) = A(t)T(t)\ , \quad T(0) = \mathbb{1}_4, \tag{S.12}$$

reads

$$T(t) = e^{\int_0^t A(u)du} = e^{\Gamma_1(t)a_1}\, e^{\Gamma_2(t)a_2}\, e^{\Gamma_3(t)a_3}, \tag{S.13}$$

with $\Gamma_k(t) := \int_0^t \gamma_k(u)du$. The matrices $a_k$ satisfy the following properties

$$a_k^2 = -a_k\ , \quad a_k^3 = a_k\ , \quad \ldots, \tag{S.14}$$

and hence

$$e^{\Gamma_k(t)a_k} = \mathbb{1} + (1 - e^{-\Gamma_k(t)})a_k =: \mathbb{1} + \chi_k(t)a_k. \tag{S.15}$$

Consequently we obtain

$$T(t) = \begin{pmatrix} \tilde{P}_0(t) & \tilde{P}_1(t) & \tilde{P}_2(t) & \tilde{P}_3(t) \\ \tilde{P}_1(t) & \tilde{P}_0(t) & \tilde{P}_3(t) & \tilde{P}_2(t) \\ \tilde{P}_2(t) & \tilde{P}_3(t) & \tilde{P}_0(t) & \tilde{P}_1(t) \\ \tilde{P}_3(t) & \tilde{P}_2(t) & \tilde{P}_1(t) & \tilde{P}_0(t) \end{pmatrix}, \tag{S.16}$$

with abbreviations (for clarity we suppress the time dependence of $\chi_k(t)$):

$$\begin{aligned} \tilde{P}_0(t) &= 1 - \frac{1}{2}(\chi_1 + \chi_2 + \chi_3 - \frac{1}{2}(\chi_1\chi_2 + \chi_1\chi_3 + \chi_2\chi_3)), \\ \tilde{P}_1(t) &= \frac{1}{2}(\chi_1 - \frac{1}{2}(\chi_1\chi_2 + \chi_1\chi_3 - \chi_2\chi_3)), \\ \tilde{P}_2(t) &= \frac{1}{2}(\chi_2 - \frac{1}{2}(\chi_1\chi_2 - \chi_1\chi_3 + \chi_2\chi_3)), \\ \tilde{P}_3(t) &= \frac{1}{2}(\chi_3 - \frac{1}{2}(-\chi_1\chi_2 + \chi_1\chi_3 + \chi_2\chi_3)). \end{aligned}$$

In our special case of Eq. (8) of the main text one finds for $T(t)$:

$$T(t) = \begin{pmatrix} P_0(t) & P_1(t) & P_1(t) & 0 \\ P_1(t) & P_0(t) & 0 & P_1(t) \\ P_1(t) & 0 & P_0(t) & P_1(t) \\ 0 & P_1(t) & P_1(t) & P_0(t) \end{pmatrix} , \tag{S.17}$$

where

$$P_0(t) = \frac{1}{2}(1+e^{-2t}) \ , \quad P_1(t) = \frac{1}{4}(1-e^{-2t}) \ . \tag{S.18}$$

Notice that for the initial condition $\vec{P}(0) = (1,0,0,0)^T$, $\tilde{P}_i(t) = P_i(t)$. Concerning equation (36) of the main text one finds for the dynamical map in 3 dimensions ($P_3(t) = 0$ for all $t$)

$$\frac{d}{dt}T'(t) = \mathbb{M}(t)T'(t) \ , \quad T'(0) = \mathbb{1}_3, \tag{S.19}$$

with

$$\mathbb{M} = \frac{1}{2}\begin{pmatrix} -2 & 2 & 2 \\ 1 & -2 & 0 \\ 1 & 0 & -2 \end{pmatrix} \tag{S.20}$$

the following solution

$$T'(t) = \begin{pmatrix} P_0(t) & 2P_1(t) & 2P_1(t) \\ P_1(t) & \frac{1}{2}[P_0(t)+e^{-t}] & \frac{1}{2}[P_0(t)-e^{-t}] \\ P_1(t) & \frac{1}{2}[P_0(t)-e^{-t}] & \frac{1}{2}[P_0(t)+e^{-t}] \end{pmatrix} . \tag{S.21}$$

Equation (31) of the main text defines a non-Markovian classical master equation whereas Eq. (36) of the main text is perfectly Markov. Both equations provide the same solution if we assume initial conditions $(1,0,0,0)^T$ for (31) and $(1,0,0)^T$ for (36).
